# Death from Anæsthetics

**Published:** 1881-11

**Authors:** 


### DEATHS FROM ANÆSTHETICS.

In the early days of chloroform, and before its dangers had been realized by the profession, a gentleman—so the story goes—applied to a dentist for the removal of a tooth under the influence of the anæsthetic. No chloroform was at hand and the dentist sallied out to get some. On his return he found his patient dead in the operating chair. Since then surgeons have, from time to time, recorded similar narrow escapes from the blame of having had patients die from anæsthetics while under operation, and it has been generally recognized that in a certain proportion of the so-called deaths from chloroform, the lethal result has been due quite as much to the mental influence of fear upon a weakened heart muscle, as to the direct action of the drug.

A little girl was brought by mother to the dental department of St. Bartholomew's Hospital. She was attended to, and, before leaving, the mother stated that the last time the child visited the hospital, her sister who accompanied her was so frightened by what she had seen that on arriving home she was seized with a fit and died almost immediately. What would have been said, we wonder, by an enlightened coroner's jury, if the elder child had chanced to have been put under gas while at the hospital.

Again, very recently, the assistant chloroformist at the same institution administered gas and ether—on Mr. Clover's plan—to a female patient, for a perineal operation, which was in every way satisfactory. A few days later the same patient was placed on the operating table with a view of judging of the success of the operation, when after a few minutes she suddenly expired. Post mortem examination revealed extensive disease of the heart.

We wish the public, with its facile *post hoc, propter hoc*, would ponder over cases like these, and learn to make some allowance for the unfortunate practitioners who have had patients die under their care.—*British Jour. Dent. Science*, Sept. 15, '81.

